# The life and legacy of Ignacio Ponseti

**Published:** 2010

**Authors:** Matthew B Dobbs, Shah Alam Khan

**Affiliations:** *Associate Professor of Orthopaedic Surgery, Washington University School of Medicine*, 1 Children's Place, Suite 4S-60, Saint Louis, MO 63110, USA E-mail: dobbsm@wudosis.wustl.edu; 1 *Associate Professor, All India Institute of Medical Sciences, New Delhi - 29, India*


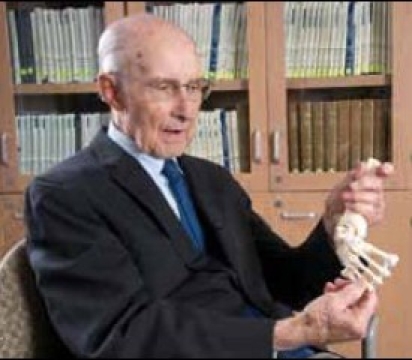


Dr. Ignacio Vives Ponseti was born in Ciutadella de Menorca, Spain, on June 3, 1914 and died in Iowa City, Iowa on October 18, 2009, at the age of 95. His legacy is the development of a primarily nonoperative method of clubfoot treatment, which involves serial casting, heel cord tenotomy, and brace wear. This method has become the gold standard of clubfoot treatment and has benefited tens of thousands patients worldwide. It could be easily concluded that Ponseti's paper on clubfoot management (1963) is one of the few manuscripts in orthopaedic literature which has changed the practice as we know it now.

Dr. Ponseti grew up in Spain where he spent his summers working for his father, a watchmaker. He entered Medical School in 1931 at the University of Barcelona- the year that the monarchy of Spain fell. He graduated in 1936 - the same time that the Spanish Civil war began. He joined the war effort and took care of fractures and wounds on the battlefield. He escaped to France in 1939 while transporting wounded soldiers. After six months he boarded a ship to Mexico as the Mexican government was offering refuge. He then practiced general medicine in rural Mexico for two years. He left for Iowa City in 1941 to train with Dr. Arthur Steindler.

His interest in clubfoot began in the early 1940s when he began his career in Iowa City at the State University of Iowa (now the University of Iowa). At that time operative management of clubfoot was popular and Ponseti found that many patients treated with extensive surgeries for clubfoot had rigid, weak and painful feet at long-term follow-up. He questioned the existence of a better treatment option for clubfoot. To answer this question, Ponseti devoted himself to furthering his understanding of the anatomy of the hindfoot, which he did, in part, through the dissection of stillborn babies. Based upon his improved understanding of clubfoot anatomy, he developed his nonoperative approach to clubfoot, which he began to utilize in 1948. In 1963, Ponseti published his early results with this method on a group of 67 clubfoot patients. A later article in which he followed the same group of patients for 10-27 years showed that the majority of the patients had flexible feet without the need for extensive surgery. Despite these results, extensive surgery continued to be performed worldwide for clubfoot correction.

It wasn't until the 1990s after Dr. Ponseti came out of retirement that his method really began to take hold. He published his book, entitled, “Congenital Clubfoot: Fundamentals of Treatment,” in 1996. His book was one of many factors during this time period that led to a renewed interest about his technique for treating clubfoot. Dr. Ponseti, in fact, credits the spread of his method mainly to clubfoot families requesting more conservative treatment from their orthopedic surgeons. Clubfoot families found a forum through use of the internet to communicate with one another. This led to families seeking surgeons who would treat their children without major surgery. Because many parents were now seeking out physicians trained in the Ponseti Method, orthopaedic surgeons began traveling to Iowa City from around the world to train with Dr. Ponseti and become proficient in his method. Now, only a decade later, the Ponseti Method is the standard of treatment worldwide. Dr. Ponseti was a remarkable physician and a shining example of the type of impact a devoted and caring physician can make. He will be greatly missed but his legacy will live on for years to come. Countless children will live normal lives without pain and disability due to his efforts.

Ponseti is survived by his wife, Helena Percas-Ponseti, whom he married in 1960 in Iowa and his son Bill Ponseti.

